# FOOD INSECURITY ACROSS THE LIFE COURSE AND COGNITIVE FUNCTION AMONG OLDER MEXICAN ADULTS

**DOI:** 10.1093/geroni/igac059.675

**Published:** 2022-12-20

**Authors:** Joseph Saenz, Jamie Kessler, Ehlana Nelson

**Affiliations:** University of Southern California, Los Angeles, California, United States; University Of Southern California, Los Angeles, California, United States; University of Southern California, Los Angeles, California, United States

## Abstract

Food insecurity is a global public health problem and is related with poorer cognition among older adults. Few have examined how food insecurity, throughout life, relates with cognition among older Mexican adults. Data came from the 2015 Mexican Health and Aging Study (n=11,507, aged 50+). Early- and late-life food insecurity were self-reported. We evaluated how food insecurity related with cognition (Verbal Learning, Verbal Recall, Visual Scanning, and Verbal Fluency), controlling for health and sociodemographic confounders. In descriptive analyses, respondents who experienced food insecurity in early- or late-life performed worse on all cognitive tasks compared to the food secure. When adjusting for health and sociodemographic confounders, early-life food insecurity predicted worse Verbal Learning performance and late-life food insecurity related with poorer Visual Scanning. Food insecurity negatively related with cognition among older Mexican adults. The significance of effects depended on cognitive task and when in life food insecurity was experienced

